# Protein acetylation: an important mechanism in actinobacteria

**DOI:** 10.1042/BSR20170851

**Published:** 2018-03-21

**Authors:** Huaidong Zhang, Ximing Xu

**Affiliations:** 1 Institute of Bioinformatics and Medical Engineering, School of Electrical and Information Engineering, Jiangsu University of Technology, Changzhou 213000, China; 2 Wuhan Institute of Virology, Chinese Academy of Sciences, Xiao Hong Shan No. 44, Wuhan 430071, China; 3 Université Paris Diderot, Sorbonne Paris Cité, Unité BFA, CNRS UMR 8251, Paris 75013, France

**Keywords:** acetyltransferase, allosteric regulation, amino acid-binding

## Abstract

This is a commentary on the research article by Lu et al. recently published in *Bioscience Reports*. The GCN5-like acetyltransferases with amino acid-binding (ACT)-GCN5-related N-acetyltransferase (GNAT) domain organization have been identified in actinobacteria by Lu et al. (2017). The ACT domain is fused to the GNAT domain, conferring amino acid-induced allosteric regulation to these protein acetyltransferases (Pat) (amino acid sensing acetyltransferase (AAPatA)). Members of the AAPatA family share similar secondary structure and are divided into two groups based on the allosteric ligands of the ACT domain: the asparagine (Asn)-activated PatA and the cysteine (Cys)-activated PatA. The former are mainly found in *Streptomyces*; the latter are distributed in other actinobacteria. The authors investigated the effect of Asn and Cys on the acetylation activity of Sven_0867 (SvePatA, from *Streptomyces venezuelae* DSM 40230) and Amir_5672 (AmiPatA, from *Actinosynnema mirum* strain DSM 43827), respectively, as well as the relationship between the structure and function of these enzymes. Research history and progress on acetyltransferases and lysine acetylation of proteins were discussed. The activity of PatA and acetylation level of proteins may be closely correlated with intracellular concentrations of Asn and Cys in actinobacteria.

Lysine acylation is a dynamic, reversible, and conserved post-translational modification (PTM). Lysine acylation and the acetyltransferase can influence several fundamental cellular pathways including cell apoptosis, cellular differentiation, and metabolism; Acyl-CoA is the donor of acylation as well as the precursor of secondary metabolism. However until recently, the significance and function of the acylation in bacteria and the archaea were obscure. The studies of lysine acylation in actinomycetes only focussed on a few species of *Streptomyces*, such as *Streptomyces coelicolor* [1]. As actinomycetes are the main source of antibiotics, further understanding of acylation regulatory mechanisms in actinomycetes will be helpful in the natural products’ production in these organisms.

Protein lysine acetylation, an important acylation form, plays a role in epigenetics, transcription, metabolism regulation, and protein–protein interaction. The recent research published by *Bioscience Reports* entitled ‘Identification and characterization of two types of amino acid-regulated acetyltransferases in actinobacteria’ [2] is, thus of great interest. This study presents a comprehensive study on identification and characterization of GCN5-like acetyltransferases with amino acid-binding (ACT)-GCN5-related N-acetyltransferase (GNAT) domain in actinobacteria. In this study, two kinds of protein acetylation mechanism were elucidated. The ACT domain fused to GNAT domain confers amino acid-induced allosteric regulation to these acetyltransferases (Pat) (amino acid sensing acetyltransferase, AAPatA). According to the allosteric ligand type of the ACT domain, members of AAPatA family are divided into two groups, the asparagine (Asn)-activated PatA and the cysteine (Cys)-activated PatA. The former exists only in *Streptomyces*; the latter are distributed in other actinobacteria (Pseudonocardiaceae, Micromonosporaceae, Nocardiopsaceae, and Streptosporangiaceae). The effect of Asn and Cys on the acetylation activity of Sven_0867 (SvePatA, from *Streptomyces venezuelae* strain DSM 40230) and Amir_5672 (AmiPatA, from *Actinosynnema mirum* strain DSM 43827) was investigated using bioinformatics and biochemistry. Lu et al. research examined the protein-acetylating acetyltransferase activity of AmiPatA and SvePatA. The activity of AmiPatA was regulated allosterically by Cys binding, while Asn was needed to regulate allosterically activity of SvePatA. Asp^16^ and Ser^17^ at the interface between β1 and α1 may somehow affect the Cys binding of AmiPatA. Nevertheless, Lys^112^ and Pro^113^ were not involved in the Asn binding of SvePatA.

Early studies focussed on the acetylation of histones and a large number of acetyltransferases and deacetylases were identified. There are three classes of histone acetyltransferases, GCN5-related N-acetyltransferase, MYST family, and CBP/p300. The GNAT family is widely distributed in eukaryotes and prokaryotes. Many groups sought homologous sequences in prokaryotes for the sake of finding similar acetyltransferases and their substrates. While the only protein *N*ε-lysine acetyltransferase (Pat) found in prokaryotes, which was discovered in *Salmonella enterica* in 2004 by Starai and Escalante-Semerena [3], regulated the activity of the central metabolic enzyme acetyl-coenzyme A synthetase (Acs). There are two families of deacetylases in eukaryotes, the Zn^2+^-dependent Rpd3/Hda1 family and the NAD^+^-dependent sirtuin family. Although many predicted homologous sequences of deacetylases in *Escherichia coli*, the only protein with deacetylase activity is CobB [4]. Sir2 is an NAD^+^-dependent deacetylase, while CobB, which is homologous to Sir2, was first studied in the ADP-ribosyltransferase function [5]. In 2002, Starai et al. [6] found that acetyl coenzyme A synthetase activity from *Salmonella enterica* was post-translationally regulated by acetylation of Lys^609^, and activation of the acetylated enzyme required the NAD-dependent protein deacetylase activity of the CobB, Sir2 protein from *S. enterica*. This was one of the first reports about the protein acetyltransferase (Pat) in prokaryotics. Then, a series of studies on CobB (deacetylase) and Pat (acetylase) in *E. coli* were carried out. Li et al. [7] found that CobB can catalyze the deacetylation of CheY, which regulated the chemotaxis of *E. coli* cells. Zhang et al. [8] successfully found nine CobB substrates using the *E. coli* protein chip. In 2010, Thao et al. [9] screened the *E. coli* proteome for substrates of the bacterial Gcn5-like Pat, and found that Pat acetylated residue Lys^180^ of RcsB (a transcription factor), and the NAD^+^-dependent Sir2 (sirtuin)-like protein deacetylase (CobB) deacetylated acetylated RcsB (RcsB(Ac)). The acetylation affected the RcsB activity, thus regulated RcsB DNA binding. It was noteworthy that Pat/CobB is a pair of acetyltransferase/deacetylase in the regulation of RcsB [9]. Yang [10] analyzed the binding domains of enzymes that are neccessary to acetylated substrates and suggested that the acetylation specificity and level were controlled by signal-dependent association of substrate with acetyltransferases and deacetylases. The bromodomain of GCN5, PCAF, TAF1, and CBP can specifically recognize acetyl lysine residues in histones, HIV Tat, p53, c-Myb, or MyoD. The flanking sequences to the acetyl lysine moiety were also involved in substrate recognition [10]. The ε-amino group of lysine residues can also be modified by methylation, ubiquitination, and sumoylation, so acetylation would exclude these PTMs. Therefore, through ‘loss-of-function’ and ‘gain-of-function’ mechanisms, acetylation may exert multifaceted effects, as exemplified by p53, whose acetylation augments DNA binding, blocks ubiquitination, and generates docking sites for bromodomain-containing co-activators [11]. Xu et al. [12] investigated the ACT-containing GNAT acetyltransferase, Micau_1670 (MaKat), from *Micromonospora aurantiaca* ATCC 27029 and found that MaKat was an amino acid-regulated Pat, whereas arginine and Cys stimulated the activity of MaKat with regard to acetylation of acetyl-CoA synthetase (Micau_0428) [12].

In the past, lysine acetylation of proteins from different microorganisms had been studied, including *E. coli, Bacillus subtilis*, and *Mycobacterium tuberculosis*. Although a large number of acetylation proteins had been identified, acetyltransferases and deacetylases in prokaryotes were still poorly understood. It was clear that lysine acetylation in prokaryotes had regulatory consequences. For example, the prokaryotes can acetylate both the α-amino groups of N-terminal residues and the ε-amino groups of lysine side chains [13]. Because of the requirement for acetyl-CoA and NAD^+^ for Pat and Sir2-type deacetylases, respectively, it may be particularly important in regulating central metabolism in prokaryotes. More evidences are required to confirm the precise metabolism pathway in prokaryotes. The activity of Pat is also controlled by allosteric effects. In *M. tuberculosis* and *Mycobacterium smegmatis*, cAMP directly activated the Pat MtKat (Rv0998) and MsKat (MSMEG_5458) by binding to a cyclic nucleotide-binding domain that was fused to the N-terminus of the catalytic GNAT domain [14]. It was likely that the Pat enzymes are carefully regulated at the transcriptional and post-translational levels in response to changes of the intracellular signals that controlled the acetylation of specific proteins, which in turn moulded the metabolic network [12]. The study by Lu et al. [2] **elucidated two kinds of protein acetylation mechanism in actinobacteria**. In their study, the authors showed that the acetyltransferase activity was allosterically regulated by ligand binding. The relationship between the structure and function of SvePatA and AmiPatA showed that some amino acid residues at the interface between β1-sheet and α1-helix may affect the ligand-binding activity. The archetypical acetyltransferases AAPatAs possessing GNAT and ACT domains showed a novel signaling pathway for regulating the acetylation of cellular proteins. The acetylation level of proteins may be closely correlated with intracellular concentrations of Asn and Cys in actinobacteria. These findings indicated that acetyltransferase activity and protein acetylation may be tightly correlated with intracellular amino acid metabolism in actinobacteria. AAPatA may acetylate enzymes involved in Asn and Cys metabolism and may regulate these pathways in response to intracellular Asn or Cys concentrations. It is helpful to understand the acetylation regulating effect on central metabolism in prokaryotes. The present study could attract more researches on the the relationship between acetylation and microbial synthesis metabolic pathway.

## Abbreviations

AAPatA: amino acid sensing acetyltransferase
ACT: GCN5-like acetyltransferase with amino acid-binding
CBP: CREB binding protein
GNAT: ACT-GCN5-related N-acetyltransferase
MYST: MOZ, Ybf2/Sas3, Sas2, and Tip60
Pat: protein acetyltransferase
PCAF: P300/CBP-associated factor
PTM: post-translational modification

## References

[1] Hesketh A.R., Chandra G., Shaw A.D., Rowland J.J., Kell D.B., Bibb M.J. (2002) Primary and secondary metabolism, and post-translational protein modifications, as portrayed by proteomic analysis of *Streptomyces coelicolor*. Mol. Microbiol. 46, 917–932 10.1046/j.1365-2958.2002.03219.x 12421300

[2] Lu Y.X., Liu X.X., Liu W.B. and Ye B.C. (2017) Identification and characterization of two types of amino acid-regulated acetyltransferases in actinobacteria. Biosci. Rep. 37, BSR20170157, 10.1042/BSR20170157 28539332 PMC6434083

[3] Starai V.J. and Escalante-Semerena J.C. (2004) Identification of the protein acetyltransferase (Pat) enzyme that acetylates acetyl-CoA synthetase in *Salmonella enterica*. J. Mol. Biol. 340, 1005–1012 10.1016/j.jmb.2004.05.010 15236963

[4] Zhao K., Chai X. and Marmorstein R. (2004) Structure and substrate binding properties of cobB, a Sir2 homolog protein deacetylase from *Escherichia coli*. J. Mol. Biol. 337, 731–741 10.1016/j.jmb.2004.01.060 15019790

[5] Frye R.A. (1999) Characterization of five human cDNAs with homology to the yeast SIR2 gene: Sir2-like proteins (sirtuins) metabolize NAD and may have protein ADP-ribosyltransferase activity. Biochem. Biophys. Res. Commun. 260, 273–279 10.1006/bbrc.1999.0897 10381378

[6] Starai V.J., Celic I., Cole R.N., Boeke J.D. and Escalante-Semerena J.C. (2002) Sir2-dependent activation of acetyl-CoA synthetase by deacetylation of active lysine. Science 298, 2390–2392 10.1126/science.1077650 12493915

[7] Li R., Gu J., Chen Y.Y., Xiao C.L., Wang L.W., Zhang Z.P. (2010) CobB regulates *Escherichia coli* chemotaxis by deacetylating the response regulator CheY. Mol. Microbiol. 76, 1162–1174 10.1111/j.1365-2958.2010.07125.x 20345663 PMC2883070

[8] Zhang Q.F., Gu J., Gong P., Wang X.D., Tu S., Bi L.J. (2013) Reversibly acetylated lysine residues play important roles in the enzymatic activity of *Escherichia coli* N-hydroxyarylamine O-acetyltransferase. FEBS J. 280, 1966–1979 10.1111/febs.12216 23452042

[9] Thao S., Chen C.S., Zhu H. and Escalante-Semerena J.C. (2010) Nepsilon-lysine acetylation of a bacterial transcription factor inhibits Its DNA-binding activity. PLoS ONE 5, e15123 10.1371/journal.pone.0015123 21217812 PMC3013089

[10] Yang X.J. (2004) Lysine acetylation and the bromodomain: a new partnership for signaling. Bioessays 26, 1076–1087 10.1002/bies.20104 15382140

[11] Liu Q., Kaneko S., Yang L., Feldman R.I., Nicosia S.V., Chen J. (2004) Aurora-A abrogation of p53 DNA binding and transactivation activity by phosphorylation of serine 215. J. Biol. Chem. 279, 52175–52182 10.1074/jbc.M406802200 15469940

[12] Xu J.Y., You D., Leng P.Q. and Ye B.C. (2014) Allosteric regulation of a protein acetyltransferase in *Micromonospora aurantiaca* by the amino acids cysteine and arginine. J. Biol. Chem. 289, 27034–27045 10.1074/jbc.M114.579078 25124041 PMC4175341

[13] Jones J.D. and O’Connor C.D. (2011) Protein acetylation in prokaryotes. Proteomics 11, 3012–3022 10.1002/pmic.201000812 21674803

[14] Nambi S., Basu N. and Visweswariah S.S. (2010) cAMP-regulated protein lysine acetylases in mycobacteria. J. Biol. Chem. 285, 24313–24323 10.1074/jbc.M110.118398 20507997 PMC2915667

